# In-Vivo LC-OCT Evaluation of the Downward Proliferation Pattern of Keratinocytes in Actinic Keratosis in Comparison with Histology: First Impressions from a Pilot Study

**DOI:** 10.3390/cancers13122856

**Published:** 2021-06-08

**Authors:** Cristel Ruini, Sandra Schuh, Charlotte Gust, Daniela Hartmann, Lars Einar French, Elke Christina Sattler, Julia Welzel

**Affiliations:** 1 Department of Dermatology and Allergy, University Hospital, LMU Munich, 80337 Munich, Germany; charlotte.gust@t-online.de (C.G.); daniela.hartmann@med.uni-muenchen.de (D.H.); lars.french@med.uni-muenchen.de (L.E.F.); elke.sattler@med.uni-muenchen.de (E.C.S.); 2 PhD School in Clinical and Experimental Medicine, University of Modena and Reggio Emilia, 41125 Modena, Italy; 3 Department of Dermatology and Allergy, University Hospital, 86156 Augsburg, Germany; Sandra.Schuh@uk-augsburg.de; 4 Dr. Phillip Frost Department of Dermatology & Cutaneous Surgery, Miller School of Medicine, University of Miami, Miami, FL 33125, USA

**Keywords:** actinic keratosis, PRO, dysplasia, keratinocyte cancer, field cancerization, line-field confocal optical coherence tomography, bedside histology, skin imaging, non-invasive diagnostics in dermatology

## Abstract

**Simple Summary:**

Actinic keratoses (AKs) are extremely common in the elderly population; they are universally recognized as precursors of invasive squamous cell carcinoma, and their risk of progression relates to the basal growth pattern of keratinocytes in the histological slides, based on a model called “PRO” classification. Since AKs can be investigated at bedside with non-invasive devices such the new line-field confocal optical coherence tomography (LC-OCT), we hypothesized that it was also possible to use such devices for reproducing the PRO classification and assessing the progression risk of AK without an invasive biopsy. In this pilot study, we demonstrated the feasibility of the LC-OCT severity grading of AKs based on the histological PRO classification, obtaining a good correlation between the two models and strong interobserver agreement.

**Abstract:**

It is known that actinic keratoses (AKs) can progress to invasive squamous cell carcinoma (SCC). The histological PRO grading of AKs is based on the growth pattern of basal keratinocytes and relates to their progression risk. AKs can be non-invasively characterized by line-field confocal optical coherence tomography (LC-OCT). The aim of the study was to define criteria for an LC-OCT grading of AKs based on the PRO classification and to correlate it with its histological counterpart. To evaluate the interobserver agreement for the LC-OCT PRO classification, fifty AKs were imaged by LC-OCT and biopsied for histopathology. PRO histological grading was assessed by an expert consensus, while two evaluator groups separately performed LC-OCT grading on vertical sections. The agreement between LC-OCT and histological PRO grading was 75% for all lesions (weighted kappa 0.66, 95% CI 0.48–0.83, *p* ≤ 0.001) and 85.4% when comparing the subgroups PRO I vs. PRO II/III (weighted kappa 0.64, 95% CI 0.40–0.88, *p* ≤ 0.001). The interobserver agreement for LC-OCT was 90% (Cohen’s kappa 0.84, 95% CI 0.71–0.91, *p* ≤ 0.001). In this pilot study, we demonstrated that LC-OCT is potentially able to classify AKs based on the basal growth pattern of keratinocytes, in-vivo reproducing the PRO classification, with strong interobserver agreement and a good correlation with histopathology.

## 1. Introduction

Actinic keratoses (AKs) or solar keratoses are scaly, pink to reddish brown macules appearing as single to multiple elements on sun-exposed areas of elderly patients’ skin. Clinically, AKs are divided into three main categories, from slightly palpable, to moderately thick, to hyperkeratotic [1].

Originally described as keratoma senilis, they were renamed by Hermann Pinkus, who combined their keratotic texture and their relationship with sunlight exposure [2]. Scalp, face, ears, neck, forearms and dorsal hands are the most involved body sites; males are more often affected than females [3]. In addition to UV radiation, other risk factors include fair skin, advanced age and chronic immunosuppression [4,5].

AKs are at risk of progressing to invasive squamous cell carcinoma (SCC) [6,7,8,9,10,11]. In fact, AKs and SCC share overlapping cytological features (atypical keratinocytes, loss of polarity, nuclear pleomorphism, altered maturation, higher mitotic index), which probably express the continuum of DNA damage due to chronic sun exposure, leading to the concept of field cancerization [12,13,14,15]. By definition, dysplastic alterations in AKs have to be confined to foci within the epidermis, while tumor strands invading the dermo-epidermal junction (DEJ) characterize invasive SCCs [16,17]. The most common histological classification divides AKs into three subgroups based on the distribution of the atypical keratinocytes within the epidermis, from the lower third (AK I), to two-thirds (AK II), to full thickness atypia (AK III) [9]. Surprisingly, this model does not correlate with progression to SCC, since the highest risk for invasion is instead associated with AKs with atypical keratinocytes restricted to the lower epidermis third (AK I). Recent studies observed that advanced basal proliferation of keratinocytes was related to progression to invasive SCC [18,19]. For this reason, a new AK histopathological classification focused on the basal growth pattern of keratinocytes was developed; the model was named “PRO I-III”, since it was focused on different stages of downward extension of basal keratinocytes, “protruding” into the papillary dermis. The earlier stage PRO I was characterized by the “crowding” of atypical keratinocytes in the basal layer, PRO II by their “budding” in round nests into the upper papillary dermis and PRO III by the “papillary sprouting”, with spikes of atypical keratinocytes protruding into the dermis and thicker than the overlying epidermis [19,20,21].

Multiple AKs often arise on sun-damaged and elderly skin, in the context of a field cancerization. Since it is not possible to predict which AKs will undergo progression into SCC through clinical examination, the whole field cancerization should be treated [22] Non-invasive diagnostic technologies such as reflectance confocal microscopy (RCM), optical coherence tomography (OCT) and line-field confocal optical coherence tomography (LC-OCT) have been successfully used to enhance the diagnostic accuracy of AKs and to characterize the field cancerization in vivo [12,23,24,25].

As LC-OCT provides a cellular resolution, we hypothesized that it should be possible to in-vivo grade the basal growth pattern of AKs based on LC-OCT images. The aim of this study was to evaluate the agreement between the LC-OCT and histological PRO classification of AKs. A secondary aim was to assess the interobserver agreement in performing the LC-OCT PRO grading.

## 2. Materials and Methods

Fifty histologically confirmed AKs of the face, imaged with LC-OCT between January 2020 and 2021 at the Departments of Dermatology of the universities of Munich and Augsburg, were analyzed in the study. The lesions belonged to 26 males and 17 females, mean age 73.8 years (CI 57;86); we included the first sequentially recruited fifty lesions with a histologically confirmed diagnosis of AK and a complete set of evaluable LC-OCT images and histopathological slides. Previously treated and/or not histologically confirmed lesions were excluded a priori from the analysis. During the selection process, 5 additional histologically confirmed AKs were excluded before performing the evaluation and were therefore not included in the panel of the fifty lesions since the quality of LC-OCT acquisition was not sufficient to analyze the required parameters (availability of only horizontal sections and not of vertical sections, artefacts such as oil bubbles). The whole lesion was imaged and analyzed with multiple LC-OCT scans, examining the epidermis, the DEJ and the dermis from the skin surface to a depth of approximately 500 μm. LC-OCT images sized 1.2 × 0.5 mm^2^ were acquired with a prototype device (DAMAE Medical, Paris), a class 1 supercontinuum laser with a central wavelength of 800 nm. Instrument and acquisition procedures have been described elsewhere [26,27]. Image acquisition was within a time frame of 0–7 days before the biopsy. The histologic specimens were 34 deep shave biopsies (with scalpel), 10 punch biopsies, 2 curettages (deep shave with ring curette) and 4 excisions. They were included in paraffin and stained with hematoxylin & eosin (H&E), according to the standard procedures, and evaluated as in daily routine examinations. Only completely evaluable tissue specimens with a vertical orientation were examined in the study.

We considered an H&E stained histological slide the gold standard. We did not perform any supplementary analysis with additional devices. PRO classification was made on both histological slides and LC-OCT vertical images based on the criteria of the original study by Schmitz et al. [21]: PRO I was defined by atypical keratinocytes crowded at basal epidermal layers (crowding), PRO II showed a slight protrusion into the upper papillary dermis with round nests of atypical keratinocytes (budding), thinner than the overlying epidermis, and PRO III displayed spikes of atypical cells thicker than the overlying epidermis (papillary sprouting). In the event that two or more patterns were present in the same image set, the higher score was chosen according to the literature [20,21]. LC-OCT images in vertical mode were evaluated, blinded to H&E slides, by a pair of dermatologists expert in non-invasive diagnostic techniques (observer round) and, separately, by a board-certified dermatopathologist/dermatologist and a dermatologist educated in dermopathology, both experts in non-invasive diagnostic techniques (consensus round). In case of discrepancy regarding the grading, the dermatopathologist set the final decision, being the most experienced in the PRO classification. Hematoxylin & Eosin (H&E) stained slides were evaluated in a consensus by a board-certified dermatopathologist expert in both classification systems and a dermatologist educated in dermatopathology, blinded to LC-OCT images. Since the PRO classification was performed on histological slides in standard vertical sections, only vertical LC-OCT images were examined, while horizontal sections were not analyzed in order to avoid confounders and bias.

Cohen’s weighted kappa coefficient with linear weights was used to calculate the correlation between LC-OCT and histopathological PRO score including confidence intervals (CI); the two cases labeled as indefinable with LC-OCT were excluded from the analysis. The correlation was also estimated into the different subgroups (PRO I and II/III). The interobserver agreement was calculated with Cohen´s kappa coefficient, also taking into account the non-definable cases. A *p*-value lower than 0.05 was considered significant [28].

The study was approved by the ethical committee of the LMU Munich (Protocol Number 17–699).

## 3. Results

### 3.1. PRO Grading

**Figure 1:**
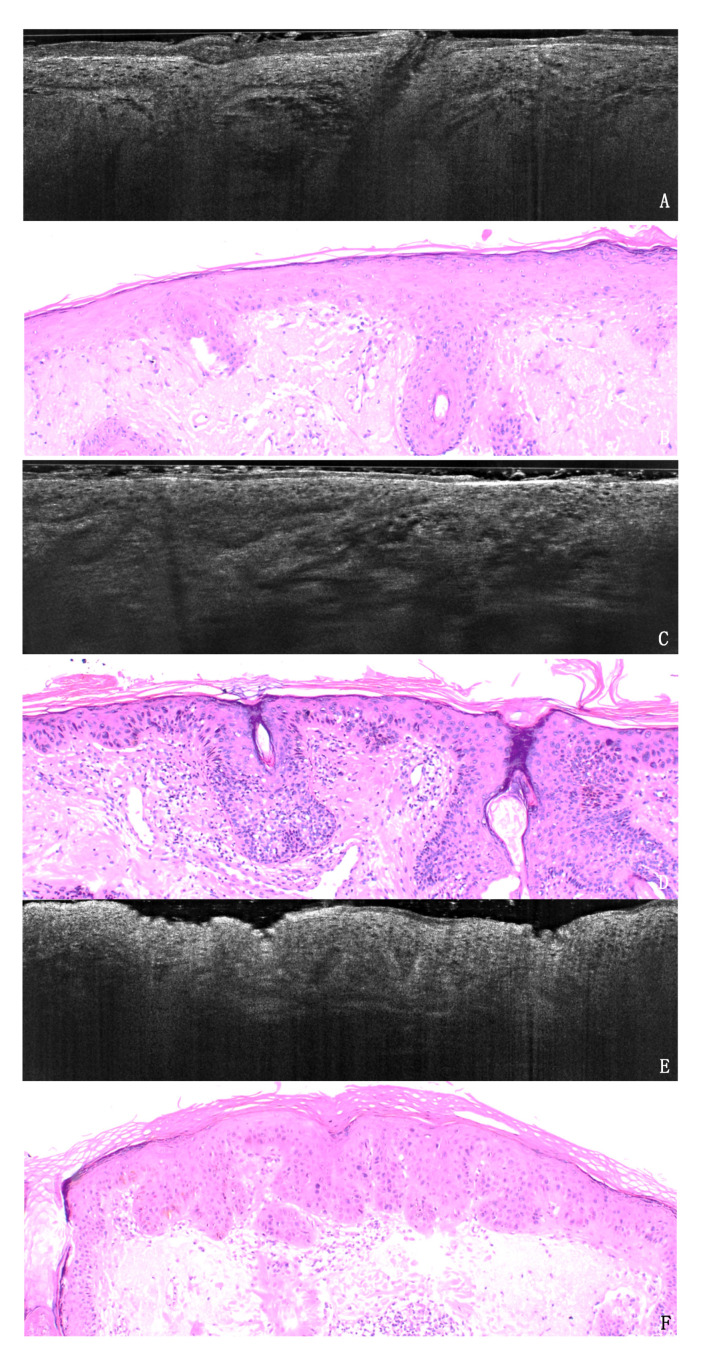
Example of three AK lesions in LC-OCT (**A**,**C**,**E**) and histology (**B**,**D**,**F**) in H&E sections, original magnification 40×. (**A**,**B**). AK PRO I shows basal crowding of keratinocytes in an otherwise regularly layered epidermis, with no protrusions into the dermis. (**C**,**D**). AK PRO II displays spikes of atypical keratinocytes slightly protruding into the papillary dermis. (**E**,**F**). AK PRO III shows the extended downward growth of atypical keratinocytes with spikes thicker than the overlying epidermis.

**Figure 2:**
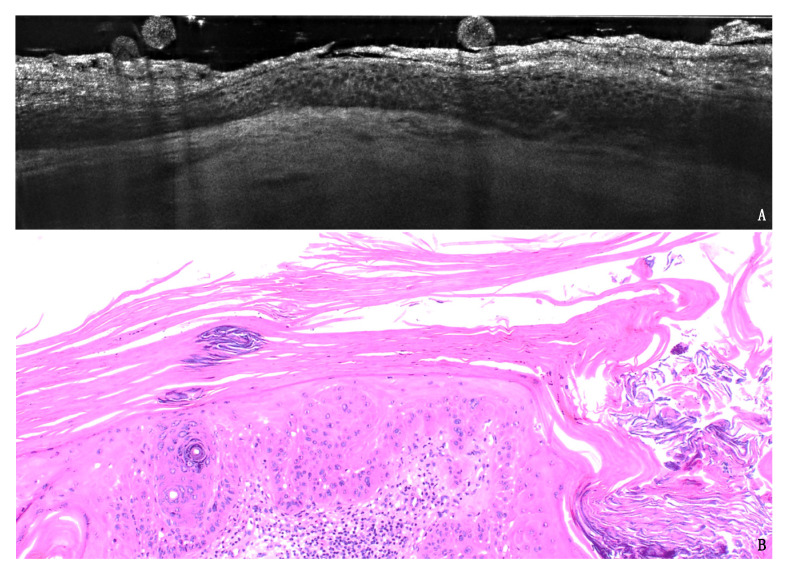
Example of AK lesion with discordant PRO grading between LC-OCT (**A**) and histology (40×, **B**): the hypertrophic AK on the scalp of an 85-year-old male patient was graded PROII in LC-OCT and PROIII in H&E, probably because of the diffuse hyperkeratoses impeding the evaluation of the deepest layers of the lesions.

Seventeen AKs were histologically classified as PRO I, 22 as PRO II and 11 as PRO III. LC-OCT PRO grading was defined as in Figure 1. The LC-OCT PRO grading was in agreement with the histological grading in 75% of the observations. The weighted kappa for LC-OCT and histological classification was 0.66 (95% CI 0.48 to 0.83; *p* ≤ 0.001). Two cases were not classifiable with LC-OCT due to excessive lesion thickness. Discordant cases were either underestimated (12%) or overestimated (12%) (Table S1, Figure 2).

**Figure 3:**
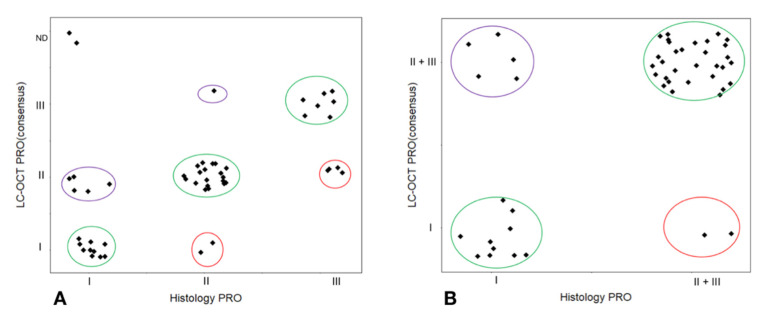
Overall (**A**) and subgroup (**B**) correlation between LC-OCT and histopathological PRO grading of AKs. ND: not definable.

If we take into account the subgroups, the agreement between LC-OCT and histology was 66.7% for PRO I, 86.5% for PRO II and 64% for PRO III. In the comparison between PRO I and PRO II-III grouped together as the higher progression risk category, the overall concordance was 85.4% (weighted kappa = 0.64, 95% CI, 0.40 to 0.88; *p* ≤ 0.001) (Figure 3).

### 3.2. Agreement

**Figure 4:**
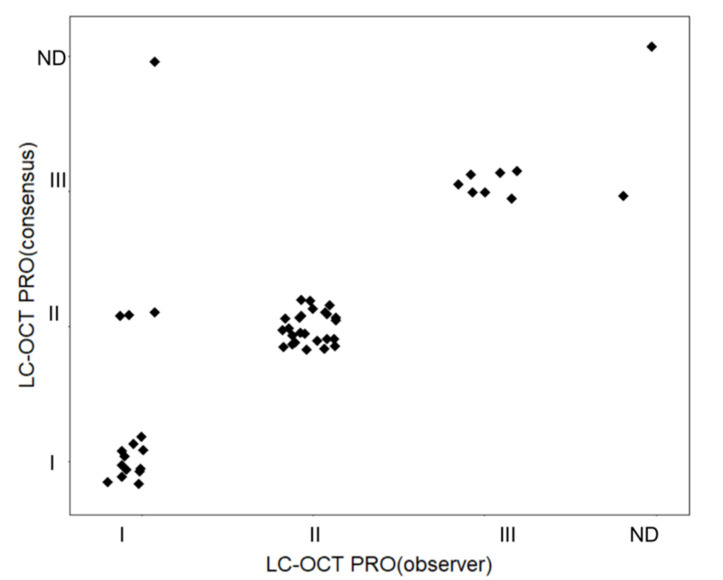
Interobserver agreement in the LC-OCT PRO classification of AKs. ND: not definable.

The interobserver agreement for LC-OCT PRO grading between the observer round and consensus round was 90%, with a Cohen´s kappa = 0.84 (95% CI 0.71 to 0.97; *p* ≤ 0.001). (Figure 4).

## 4. Discussion

AKs are one of the most common skin conditions in the elderly population, mainly arising on sun-exposed areas of the skin in the context of a field cancerization. Following the current guidelines, all AKs should be treated, since it is not possible to predict which ones will grow into an invasive SCC [29]. The type of treatment should be determined based on the clinical features, the patients’ comorbidities and the previous therapies. Various local and field treatments, including surgery, laser ablation, photodynamic therapy and diverse topical treatments such as 5-fluorouracile, diclofenac, imiquimod and potassium hydroxide, can be used. It is known that diagnosis and follow-up of AKs can be improved by non-invasive diagnostic technologies such as RCM, OCT and LC-OCT, which provide a painless bedside examination of keratinocyte cancer (KC) [12,15,24,25,30,31,32,33]. RCM has been used for grading increasing keratinocyte dysplasia into different levels, with good correlation with histopathology, but it does not refer to specific classification systems [23].

We therefore focused on the in-vivo evaluation of the growth pattern of basal keratinocytes through LC-OCT, based on the recent PRO classification. The latter proved, in fact, to ensure the best interobserver agreement and the best risk stratification (in terms of progression risk to SCC) compared to other AK classifications [19,20,21]. We chose to use LC-OCT since it delivers dermoscopy-guided and real-time cross-sectional imaging of skin lesions with cellular resolution, so that the cytological and architectural features of the epidermis can be displayed in real time. It can visualize the epidermal layers, the DEJ and the superficial dermis, and its vertical acquisitions can be intuitively compared to histological slides [30,34,35]. Moreover, additional features such as perifollicular proliferation and adnexal extension can be visible, up to a depth of 500 µm. A direct and practical consequence is the potential in-vivo assessment of AK progression risk, since PRO III AKs are associated with a higher risk of developing an invasive SCC compared to PRO I [19,20,21].

In our study, we were able to visualize and grade the downward extension of AKs non-invasively through LC-OCT, defining key imaging patterns that can be used as reference images. We reported strong interobserver agreement in grading the basal growth pattern of keratinocytes in the examined AKs. Moreover, we reported good agreement in the PRO classification of AKs between LC-OCT and traditional histopathology. Additionally, we reached an overall agreement of 85.4% when comparing the subgroup of PRO I and the subgroup of PRO II/III AKs, which are the lesions at higher risk of progression to SCC compared to PRO I.

**Figure 5:**
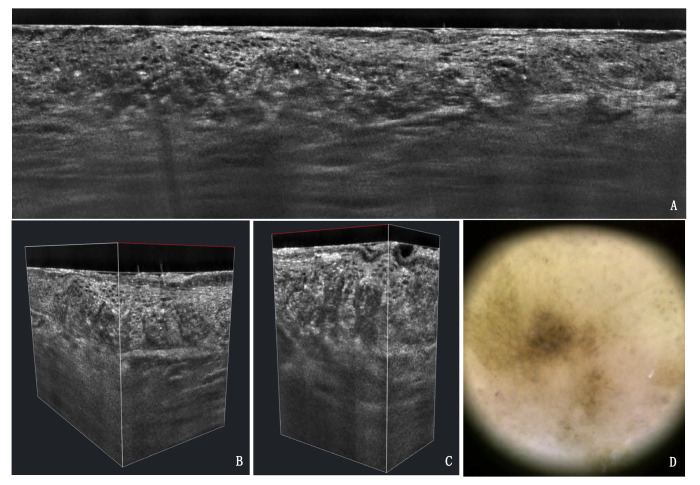
LC-OCT vertical (**A**), 3D (**B**,**C**) and dermoscopy (**D**) of a pigmented PRO III AK lesion on the dorsum of the hand of an 82-year-old male patient. The typical papillary sprouting as well as the pigmented basal keratinocytes are visible.

LC-OCT displays the protrusions of atypical basal keratinocytes into the upper dermis (spikes) and allows the measurement of their thickness compared to the overlying epidermis. This visual evaluation permits a reproducible LC-OCT grading of AKs based on the PRO histological classification. We were able to classify PRO I as lesions with no visible spikes, PRO II as lesions with spikes slightly protruding into the papillary dermis and PRO III as lesions with more prominent spikes, thicker than the overlying epidermis (Figure 1, Figure 5). Our criteria can be used as a reference model for LC-OCT grading of AKs. The high reproducibility (90%) between observers demonstrates that skilled imaging experts are able to perform an in-vivo evaluation of cyto-architectural atypia with LC-OCT.

LC-OCT can be therefore considered a useful tool for the diagnosis and therapeutic monitoring of AKs and might be successfully used in both clinical trials and daily routine. As OCT and RCM, it can be used for a live, in-vivo mapping of several lesions in different progression stages and perilesional skin in the context of field cancerization, avoiding multiple surgical biopsies and related consequences (pain, discomfort, scars, inflammatory reactions, failed localization). Compared to conventional OCT, LC-OCT has a higher resolution and allows the assessment of cytological features and not only structural details. Compared to RCM, LC-OCT displays vertical sections simultaneously to horizontal slides as in RCM. Thus, it is much easier in LC-OCT images to determine the degree of basal proliferation and compare it with histology.

Some limitations have to be taken into account. In our study, two large and thick hyperkeratotic lesions were not fully evaluable. This sheds light on the good but still not perfect penetration depth of the LC-OCT, which does not fully display the basal layer in the presence of severe hyperkeratosis. In such cases, it is useful to examine the margins of the lesion, since the basal growth pattern of keratinocytes does not depend on the lesion thickness. Moreover, in this regard, it was easier for the observers to evaluate the downward growth pattern of basal keratinocytes in the context of a PRO classification, compared to the distribution of the atypical keratinocytes through the epidermis, as in other classifications. Another limitation of the study was the small sample size (50 lesions, belonging to 43 patients). Since some lesions arose on the same patient, a bias derived from this aspect cannot be excluded; however, we only included independent lesions, arising on different anatomical sites and separately imaged and biopsied, so that we considered the number of cases acceptable for a lesion-centered, pilot study [36]. An additional, crucial point that has to be taken into account is represented by the observer´s experience, not only in non-invasive diagnostic techniques but also in dermatopathology. All observers in this study had knowledge of dermatopathology and non-invasive imaging; it was not possible to perform a correlation of the LC-OCT grading and the level of experience of the observer.

The agreement between LC-OCT and histology was lower in PRO I compared to PRO II/III, due to overestimation. Five cases graded as PRO I in histology were evaluated as PRO II/III with LC-OCT. This might be due to confounders such as hair follicles and shafts, which might appear as longitudinal structures in the sections, but also to the larger field of view, providing a global analysis of the examined area. LC-OCT can, in fact, scan the whole AK lesion in a three-dimensional way, guided by a camera, displaying multiple scans; on the contrary, the pathologist only has a few histological sections at their disposal, since only a few slides are obtained from each tissue block in the daily diagnostic routine. From this perspective, LC-OCT might provide additional details regarding non-invasive AK grading, correctly directing lesions to either conservative or surgical therapy based on their basal growth pattern.

## 5. Conclusions

LC-OCT is able to perform a reliable in-vivo evaluation of basal keratinocyte growth pattern based on the histopathological PRO classification of AKs, with strong agreement with histopathology and between different observers. Larger studies, also potentially aided by artificial intelligence algorithms, are needed to validate the method.

## Data Availability

Fully anonymized data are available on request.

## Supplementary Materials

The following are available online at https://www.mdpi.com/article/10.3390/cancers13122856/s1, Table S1: PRO grading evaluated with LC-OCT in the observer round, consensus round and histopathology.
