# Supplementary material for: In-Vivo LC-OCT Evaluation of the Downward Proliferation Pattern of Keratinocytes in Actinic Keratosis in Comparison with Histology: First Impressions from a Pilot Study
Source: Cancers (Basel). 2021 Jun 8;13(12):2856. doi: 10.3390/cancers13122856 (PMC8228287; doi:10.3390/cancers13122856)
Supplement: Supplementary file 1 [file cancers-13-02856-s001.zip › cancers-1176621-supplementary.pdf]

cancers

MDPI

In-Vivo LC-OCT Evaluation of the Downward Proliferation Pattern of Keratinocytes in Actinic Keratosis in Comparison with Histology: First Impressions from a Pilot Study

Cristel Ruini, Sandra Schuh, Charlotte Gust, Daniela Hartmann, Lars Einar French, Elke Christina Sattler and Julia Welzel

Table S1. PRO grading evaluated with LC-OCT in the observer round, consensus round and histopathology (hematoxylin-eosin stained slides) (1= PRO Grade I, 2 = PRO Grade II, 3 = PRO Grade III, 4 = PRO Grade not definable).

Lesion ID	LC-OTC PRO Grade-Observer Round	LC-OTC PRO Grade-Consensus Round	HISTOLOGY PRO Grade-Gold Stand
1	2	2	2
2	1	2	1
3	2	2	2
4	2	2	2
5	1	1	2
6	1	1	1
7	2	2	2
8	2	2	2
9	1	1	1
10	2	2	2
11	2	2	3
12	1	1	1
13	3	3	3
14	1	1	1
15	4	4	1
16	1	1	1
17	2	2	2
18	2	2	2
19	1	1	1
20	2	4	1
21	3	3	3
22	3	3	3
23	4	3	3
24	2	2	1
25	2	2	2
26	3	3	3
27	2	2	2
28	2	2	2
29	1	2	1
30	1	1	1
31	2	2	2
32	2	2	2
33	2	2	2
34	2	2	3
35	2	2	2
36	2	2	2
37	1	1	1
38	2	2	3
39	1	1	2
40	2	2	3

41	2	2	2
42	3	3	3
43	1	2	1
44	1	1	1
45	2	2	2
46	2	2	2
47	2	2	1
48	1	1	1
49	3	3	3
50	3	3	2
